# Is switching intravesical chemotherapeutic agents beneficial in short-term recurrent high-risk non-muscle-invasive bladder tumors? A 5-year retrospective study

**DOI:** 10.1186/s12894-024-01410-1

**Published:** 2024-01-31

**Authors:** Shuaiqi Chen, Guangyu Sun, Xiaoxu Chen, Tiyara Salgado, Shangrong Wu, Hailong Hu, Ranlu Liu, Yunkai Qie

**Affiliations:** 1 Department of Urology, Tianjin Institute of Urology, The Second Hospital of Tianjin Medical University, Tianjin, China; 2 Department of Urology, Tianjin Medical University General Hospital, Tianjin, China

**Keywords:** Instillation therapy, Intravesical chemotherapy, Drug switching, Non-muscle-invasive bladder cancer, RFS, PFS

## Abstract

**Objective:**

To explore if switching intravesical chemotherapeutic agents is beneficial in short-term recurrences of high-risk non-muscle-invasive bladder cancer (NMIBC) following the failure of preceding intravesical therapy.

**Materials and methods:**

From June 2010 to October 2015, 205 patients with NMIBC who experienced tumor recurrence within a year after receiving first-line intravesical chemotherapy (IVC) were classified into two groups. After a second complete transurethral resection (TUR) process, we immediately altered the intravesical instillation agent for 107 patients (group A). In contrast, the remaining 98 patients (group B) continued using their original intravesical instillation agent. After transurethral resection of the bladder tumor (TURBT), all patients received either an immediate instillation of epirubicin (EPI), gemcitabine (GEM), or hydroxycamptothecin (HCPT), followed by regular induction and maintenance instillations. Recurrence and progression rates were evaluated using the Chi-square test, and recurrence-free survival (RFS) and progression-free survival (PFS) were calculated using the Kaplan–Meier method.

**Results:**

In this study, there was no significant difference in either the 5-year tumor recurrence or progression rates between the two groups (*p* > 0.05) The Kaplan–Meier plot showed no difference in progression-free or recurrence-free survival between the two groups.

**Conclusion:**

Switching IVC agents does not improve RFS and PFS for patients with short-term recurrent high-risk NMIBC.

**Supplementary Information:**

The online version contains supplementary material available at 10.1186/s12894-024-01410-1.

## Introduction

Bladder cancer is one of the ten most prevalent cancers worldwide, with approximately 550,000 new cases yearly [1]. In China, 85,594 new cases of bladder cancer were diagnosed in 2020, with over 75% being non-muscle-invasive bladder cancer (NMIBC) and the majority being histologically low grade [2, 3]. Transurethral resection (TUR) surgery and adjuvant therapy with intravesical chemotherapy (IVC) or immunotherapy continue to be the preferred therapy for NMIBC [4–7]. Nevertheless, effective management remains challenging, as approximately 33% of patients experience recurrence within 12 months following post-resection IVC [8].

Multiple studies have demonstrated that primary or acquired drug resistance in bladder cancer cells frequently results in chemotherapy failure and tumor recurrence [9, 10]. Using drugs with distinct mechanisms of action may overcome resistance to chemotherapy for tumors, and cross-resistance is unlikely, whether used alone or in combination [11–13]. Before the Chinese Food and Drug Administration (FDA) approved Bacillus Calmette-Guérin (BCG) for the treatment of bladder cancer, our practice consisted of switching intravesical chemotherapeutic agents for short-term recurrent NMIBC. We hypothesized that switching intravesical chemotherapeutic agents after the failure of short-term IVC would provide a substantial proportion of patients with respite from recurrence and progression. This study aims to ascertain the efficacy of switching IVC drugs in patients with short-term recurrent NMIBC at high risk.

## Materials and methods

### Patients

This retrospective study examined a consecutive series of patients who had previously been diagnosed with NMIBC and who experienced a relapse within a year of their initial TUR and IVC treatments between June 2010 and October 2015 in the Second Hospital of Tianjin Medical University. After tumor recurrence and re-treatment with transurethral resection of the bladder tumor (TURBT), we immediately altered the intravesical instillation agent for group A patients, while group B patients persisted in using their initial medication. After TURBT, all patients received an immediate instillation of epirubicin (EPI), gemcitabine (GEM) or hydroxycamptothecin (HCPT), followed by regular induction and maintenance instillations. Inclusion criteria included the following: (1)Patients who underwent complete TURBT in our hospital, and postoperative pathology confirmed high-risk NMIBC; (2) all patients had their first recurrence within one year after TURBT and IVC; (3) the perfusion drug is either HCPT, GEM, or EPI, and the patient received bladder cancer medication as prescribed following surgery; (4) unavailability or unsuitability for BCG instillation, refused or were not candidates for cystectomy. Exclusion criteria included the following: (1) Tumor residual after TURBT; (2) cases with a previous or simultaneous diagnosis of upper-tract urothelial carcinoma (UTUC); (3) patients were treated with other antitumor treatments except HCPT, GEM or EPI; (4) presence of malignant tumors or serious diseases; (5) case data are incomplete during follow-up.

### Intravesical chemotherapeutic treatment protocol

All patients received an immediate instillation within 24 h after initial complete TURBT. IVC induction was subsequently performed weekly for eight weeks. The subsequent maintenance treatment comprised ten monthly treatments until the tumor recurred. Patients in group A had their IVC drugs changed following a second TURBT, while patients in group B did not. Patients in both groups were treated according to the perfusion protocol described previously. The bladder contained HCPT (40 mg/40 mL NS), GEM (1000 mg/50 mL NS), and Epirubicin (50 mg/40 mL NS) for 120, 60, and 60 min, respectively. Before instillation, the patient was instructed to empty their bladder and refrain from excessive drinking, fluids, or diuretics for two hours. The patient was asked to roll and alternate positions throughout the instillation to maximize the affected bladder wall contact.

### Follow‑up and outcomes

Pathological analysis was conducted following the initial complete TURBT to verify the stage and grade, and cystoscopy was performed every three months. A bladder biopsy was conducted to confirm the diagnosis after a cystoscopy revealed a suspicious tumor. After a second TURBT, a second pathological examination was conducted to corroborate the stage and grade. Cystoscopy was performed every three months for two years and then every six months for the following three years. The postoperative tumor recurrence and adverse reactions of drugs were identified and documented using the patient's hospital visit records (including outpatient and inpatient care) and follow-up. Recurrence was defined as any recurrence or metastasis of an intravesical tumor. As described in the references, tumor progression was defined as the recurrence of a tumor at a higher stage or grade or deterioration of disease compared to the second pathological analysis [14]. Follow-up time was defined as the time from the second TURBT to the tumor progression, last follow‐up, or end of study. The diagnosis of high-risk bladder tumors is based on either of the two pathology reports. High-risk tumors were defined as including any of the following: (1) T1 tumor, (2) high-grade tumor, (3) carcinoma in situ (CIS), and (4) multiple, recurrent, and large (> 3 cm) TaG1G2/LG tumors (all features must be present) [5, 15]. After the second TURBT, adverse reactions to IVC drugs were assessed on the first day of each cycle using the Common Terminology Criteria for Adverse Events (CTCAE) Version 4.0.

### Statistical analysis

All data in this research were analyzed using the statistical analysis software SPSS23.0, where all tests were two-sided and a p-value of less than 0.05 was considered statistically significant. We evaluated the recurrence rate, progression rate, overall survival, latency to second recurrence, and progression. The Kaplan–Meier method determined the second recurrence and progression curves. The patients' characteristics of groups A and B were compared using the Student t-test for continuous parameters and the Chi-square test for categorical parameters on independent samples. Statistical source data for Tables 1, 2 and 3 are available in Supplementary Tables 1-3, respectively.

**Table 1:** Patient and primary tumor characteristics

	Group A *n *= 107	Group B *n* = 98	*p*-value
Age medium (range years)	66 (32–89)	65 (25–86)	0.601
Sex (n%)			0.869
Male	84 (78.5)	76 (77.6)	
Female	23 (21.5)	22 (22.4)	
T category (n%)			0.393
Ta	26 (24.3)	29 (29.6)	
T1	81 (75.7)	69 (70.4)	
Grade (n%)			0.402
Low grade	44 (41.1)	46 (46.9)	
High grade	63 (58.9)	52 (53.1)	
CIS (n%)			0.248
No	104 (97.2)	98 (100)	
YES	3 (2.8)	0 (0)	
Tumor size (n%)^a^			0.358
< 3 cm	77 (72.0)	76 (77.6)	
≥ 3 cm	30 (28.0)	22 (22.4)	
Multifocal (n%)			0.527
No	38 (35.5)	39 (39.8)	
YES	69 (64.5)	59 (60.2)	

^a^Maximum size of largest tumor resected

## Results

### Patients

**Fig. 1:**
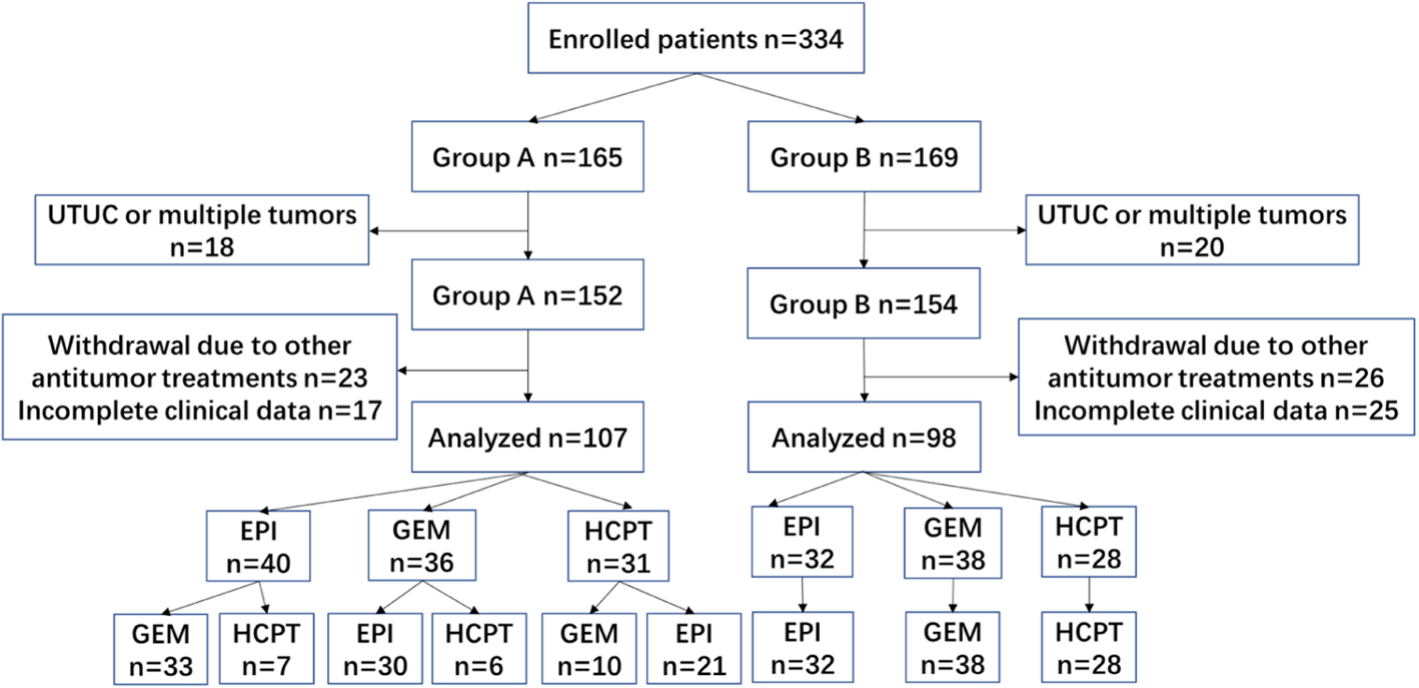
Flow diagram of this study. *UTUC* upper-tract urothelial carcinoma, *EPI* epirubicin, *GEM* gemcitabine, *HCPT* hydroxycamptothecin

A total of 334 patients were initially evaluated, and 129 patients were excluded based on our criteria. The remaining 205 patients comprised 107 in Group A and 98 in Group B. Figure 1 depicts the number of patients and the details of the instillation medications used. In Tables 1 and 2, patient characteristics are described in detail. Other statistically significant parameters were unidentified.

**Table 2:** Clinical features of recurrent tumors

	Group A	Group B	*p*-value
T category (n%)			0.052
Ta	36 (33.6)	46 (46.9)	
T1	71 (66.4)	52 (53.1)	
Grade (n%) (WHO 2004)			0.258
Low grade	44 (41.1)	48 (49.0)	
High grade	63 (58.9)	50 (51.0)	
CIS (n%)			0.600
No	105 (98.1)	94 (95.9)	
YES	2 (1.9)	4 (4.1)	
Tumor size (n%)^a^			0.815
< 3 cm	102 (95.3)	95 (96.9)	
≥ 3 cm	5 (4.7)	3 (3.1)	
Multifocal (n%)			0.072
No	41 (38.3)	26 (26.5)	
YES	66 (61.7)	72 (73.5)	

^a^Maximum size of largest tumor resected

### Efficacy

**Fig. 2:**
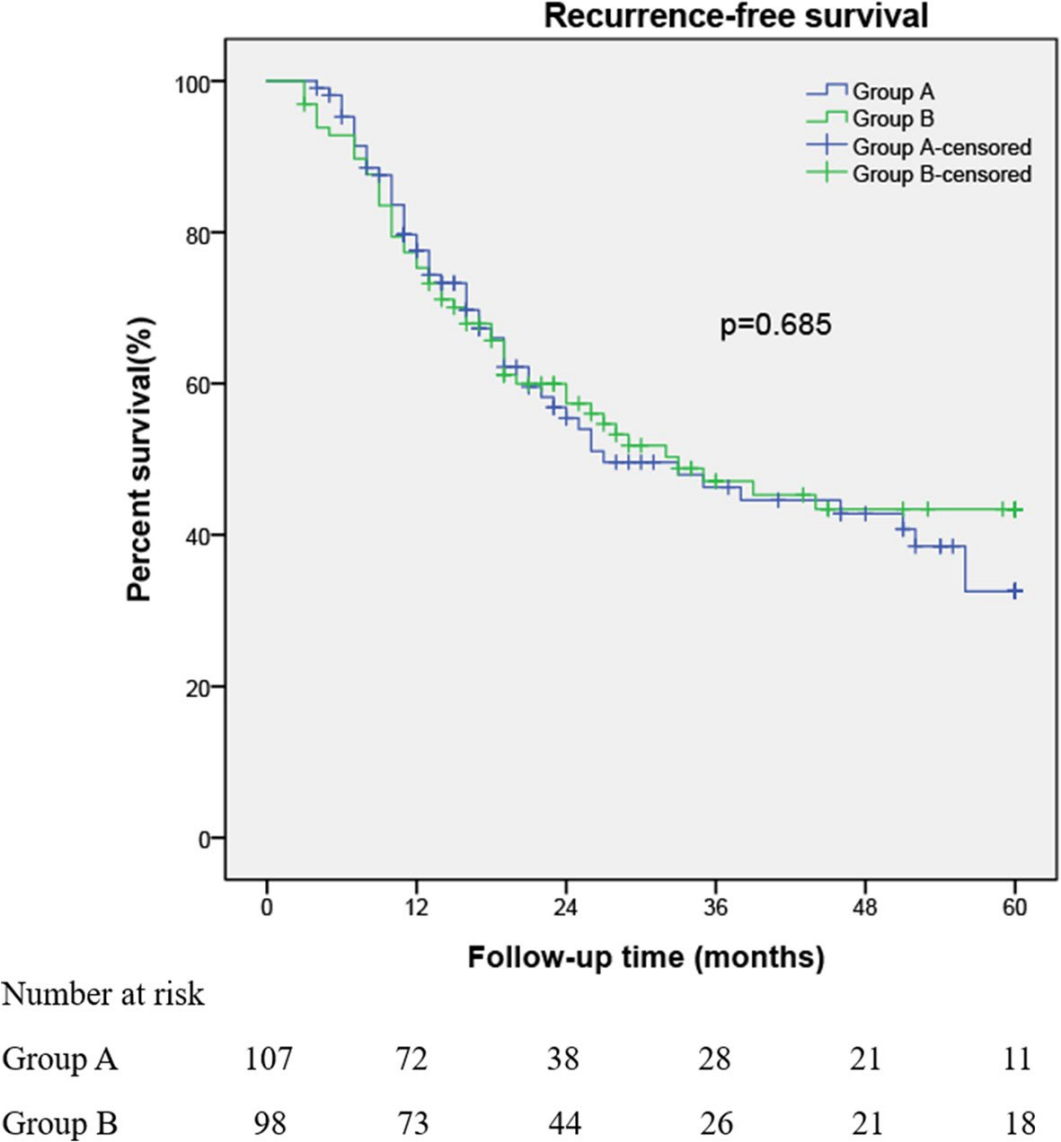
Kaplan–Meier curves for 5-year recurrence-free survival are shown. There was no statistically significant difference between the two groups' Kaplan–Meier curves for recurrence-free survival

**Fig. 3:**
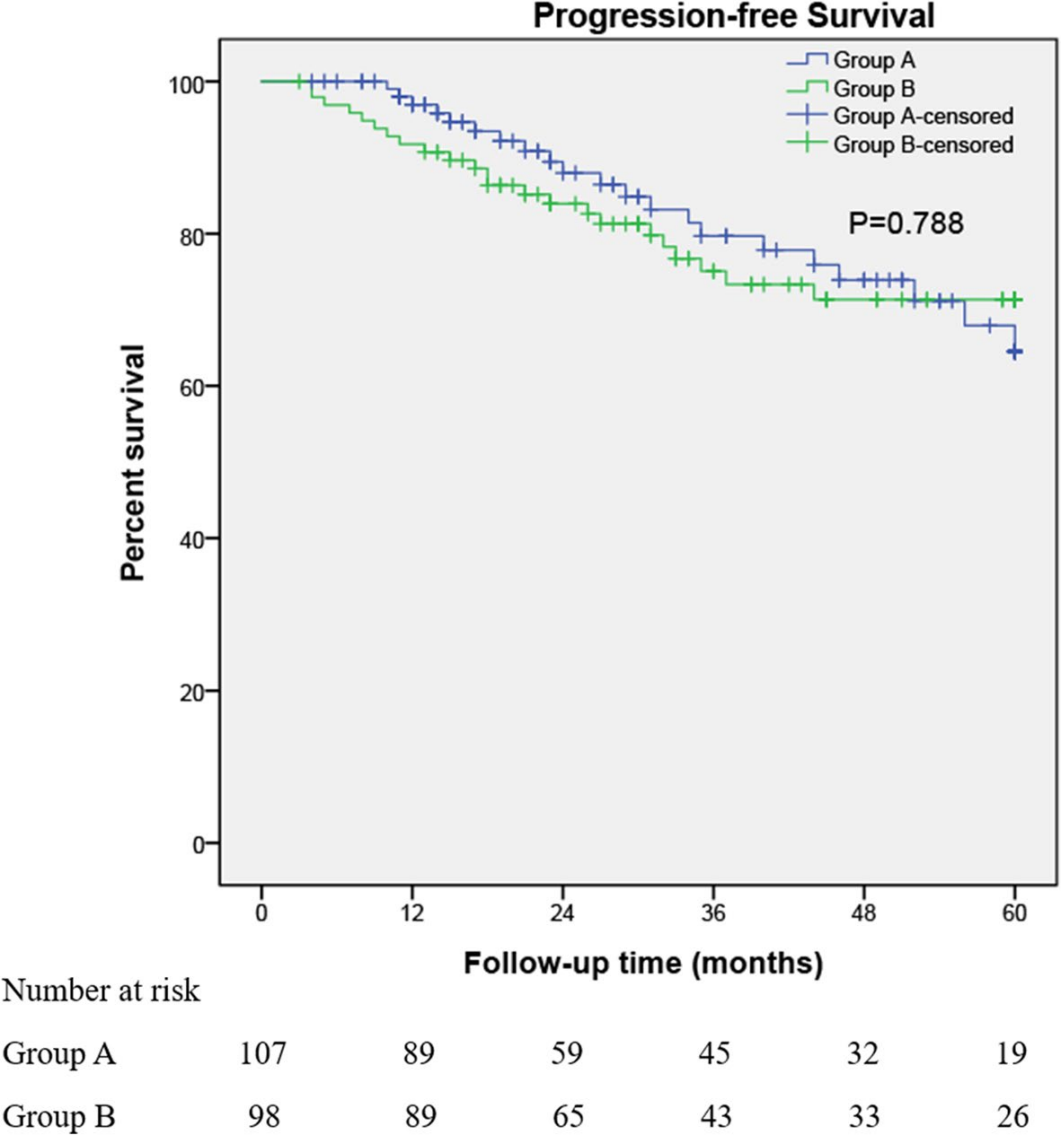
Kaplan–Meier curves of 5-year progression-free survival are shown. There was no statistically significant difference between the two groups' Kaplan–Meier curves for progression-free survival

For Group A versus Group B, median follow-up was 29 months (interquartile range [IQR]: 15.0–52.0) versus 33 months (IQR: 19–60) (*p* = 0.257), respectively. The 5-yr tumor recurrence rates between groups A and B were 49.5% (53/107) and 50.0% (49/98), respectively (*p* = 0.947). The 1-yr, 3-yr, 5-yr RFS was 21.5% versus 24.5% (*p* = 0.568), 43.9% versus 48.0% (*p* = 0.991), 49.5% versus 50% (*p* = 0.685), respectively. The 5-yr tumor progression rates between groups A and B were 18.7% (21/107) and 23.5% (23/98), respectively (*p* = 0.503). The 1-yr, 3-yr, 5-yr PFS was 3.7% versus 8.2% (*p* = 0.165), 14.0% versus 21.4% (*p* = 0.326), 19.6% versus 23.5% (*p* = 0.788), respectively. In Group A, four patients died of bladder cancer. In Group B, three patients died of bladder cancer. Kaplan–Meier curves for RFS and PFS are shown in Figures 2 and 3, of which both outcomes were not statistically associated with treatment strategies.

The adverse effects of the three IVC medications are summarized in Table 3. All three IVC medications were generally safe and well-tolerated, with no Grade 3 or higher adverse effects observed. The incidence of adverse events was 48.2% (40/83) in EPI, 35.8% (29/81) in GEM, and 36.6% (15/41) in HCPT. Overall, there were no statistically significant differences in the adverse effects among the three medications (*p* = 0.175).

**Table 3:** Incidence of adverse effects by drugs

Symptom	EPI(*n* = 83)	GEM(*n* = 81)	HCPT(*n* = 41)	
	Grade I–II	Grade I–II	Grade I–II	*P* value
Dysuria (n%)	5(6.0)	4(4.9)	2(4.9)	
Frequency (n%)	8 (9.6)	7(8.6)	4(9.8)	
Urgency (n%)	7(8.4)	6 (7.4)	3(7.3)	
Hematuria (n%)	14(16.9)	8 (9.9)	4(9.8)	
Fever (n%)	3(3.6)	2(2.5)	1(2.4)	
Chemical cystitis (n%)	2(2.4)	1(1.2)	1(2.4)	
Nausea and gvomitin (n%)	1(1.2)	0 (0)	0(0)	
Total (n%)	40(48.2)	28(34.6)	15(36.6)	0.175

*EPI* epirubicin, *GEM* gemcitabine, *HCPT* hydroxycamptothecin

## Discussion

Since the US FDA approval of BCG, various guidelines recommend the use of BCG vaccine as the standard adjunctive therapy for intermediate- and high-risk NMIBC, and as an option for intermediate-risk NMIBC, particularly after Mitomycin C (MMC) failure [5–7]. BCG therapy is approximately 27 and 32% more efficacious in reducing the risk of tumor progression and recurrence, respectively [16, 17]. However, there are numerous issues with BCG immunotherapy administered intavesically in China. First, the recent global deficit of BCG has prevented its use in most Chinese hospitals [18, 19]. Second, BCG efficacy remains suboptimal (up to 40%failure rate at two years) [20, 21]. Third, BCG vaccine therapy is associated with significantly higher post-treatment toxicity and adverse effects than IVC [7]. Furthermore, it is not covered by basic medical insurance in China and is often too expensive for patients to afford (over $ 10,000 in 1 year) [19]. Before the approval of BCG by the China FDA in 2015, IVC was the treatment of choice for preventing the recurrence of NMIBC after surgery [15]. Therefore, the BCG vaccine was not included in the scope of this study.

Even with induction and maintaining IVC following TURBT, many patients will experience NMIBC recurrence in the short term. If the BCG is unavailability or inappropriate, for this apparent treatment resistance, the Canadian Urological Association recommends single-agent chemotherapy (e.g., Mitomycin-C, Gem) or sequential combination of IVC (e.g., Gem /docetaxel) with induction followed by monthly maintenance for up to a year [13, 22]. McElree et al. reported retrospective results from a single center that treated 75 patients with sequential intravesical valrubicin and docetaxel as salvage therapy for recurrent NMIBC after the failure of intravesical BCG or Gem-docetaxel. The recurrence-free survival rate in low- and high-grade disease was 73% and 38%, respectively. Among patients with high-grade disease, overall, cancer-specific, and cystectomy-free survivals were 87, 96, and 84% at two years, respectively [23]. Due to cumulative, synergistic, or distinct antitumor mechanisms of action, combination chemotherapy may reduce drug resistance and further decrease the recurrence rate, however, it may also increase local toxicity [13, 24]. Chen et al. administered a regimen of MMC, doxorubicin, and cisplatin (MDP) to newly diagnosed papillary NMIBC patients [25]. Compared to the doxorubicin maintenance group, the MDP group had a substantially lower recurrence rate yet a higher discontinuation rate due to adverse events.

Single-agent IVC has been the standard salvage therapy and the basis for future combination therapy trials [26]. In a retrospective study, Gem or the original IVC was administered to 72 patients with recurrent NMIBC following the failure of prior intravesical therapy (MMC, EPB, and CPT) [27]. The study showed that Gem is more effective than the original chemotherapy in 2-year tumor-free survival rates (70.8% vs. 45.8%). However, the authors did not specify whether switching IVC drugs after IVC failure is beneficial in NMIBC.

To prevent tumor recurrence after TURBT for NMIBC, HCPT, GEM, and EPI, which have different antitumor mechanisms, are widely used as first-line drugs for IVC [7, 15]. To our knowledge, this is the first single-centered retrospective observational study comparing the efficacy of switching or retaining the original chemotherapy drug in this selected subset of patients with NMIBC, failing first-line drugs for adjuvant IVC, and for whom unavailability or unsuitability for BCG instillation. In the current study, the recurrence rate of Group A was 49.5%, similar to van Rhijn et al. [28]. Group A showed no significant advantages over Group B regarding recurrence rate. The progression rates in the two groups were 18.7 vs. 23.5%, and although the association was not statistically significant (*p*>0.05), the variations were considerable, similar to Cambier et al.'s finding of 19.8% [29]. Kaplan-Meier analysis revealed no significant differences in RFS or PFS between the two groups, suggesting that switching drugs in the treatment of short-term recurrent high-risk NMIBC cannot improve RFS and PFS.

The adverse effects of the three IVC medications were mild and brief, and were limited to grade two. A total of 41.0% of the patients suffered from adverse events in this study, and the most common adverse events were bladder irritation symptoms. In the group of patients receiving epirubicin, dysuria, frequency, urgency, haematuria, fever, chemical cystitis, nausea, and vomiting were higher than in the other two medications. Overall, there were no statistically significant differences in the adverse effects among the three medications (*p* = 0.175).

The current study had several limitations. Firstly, it should be acknowledged that this study lacked a prospective randomized design, posing a potential risk of selection bias despite no significant differences in patient or tumor characteristics among groups.

Secondly, this study does not address which IVC medication was most effective in preventing recurrence or progression. Thirdly, The absence of comparison data with an untreated control group is a significant limitation that should be acknowledged.

## Conclusions

In summary, compared to retaining the original IVC agent, switching IVC agents does not improve RFS and PFS for patients with short-term recurrent NMIBC at high risk. Therefore, seeking effective drugs and novel treatment concepts for those BCG-unavailable patients is required in the future.

### Supplementary Information

**Additional file 1:** **Table S1.** Statistical source data for table 1.

**Additional file 2:** **Table S2.** Statistical source data for table 2.

**Additional file 3:** **Table S3.** Statistical source data for table 3.

## Data Availability

The data and materials presented in this study are available upon request from the corresponding author.
